# Supplementary material for: Gene Copy Number Dictates Extracellular Vesicle Cargo
Source: Int J Mol Sci. 2025 Jun 8;26(12):5496. doi: 10.3390/ijms26125496 (PMC12192956; doi:10.3390/ijms26125496)
Supplement: Supplementary file 1 [file ijms-26-05496-s001.zip › ijms-3629104-supplementary.pdf]

Supplemental Information

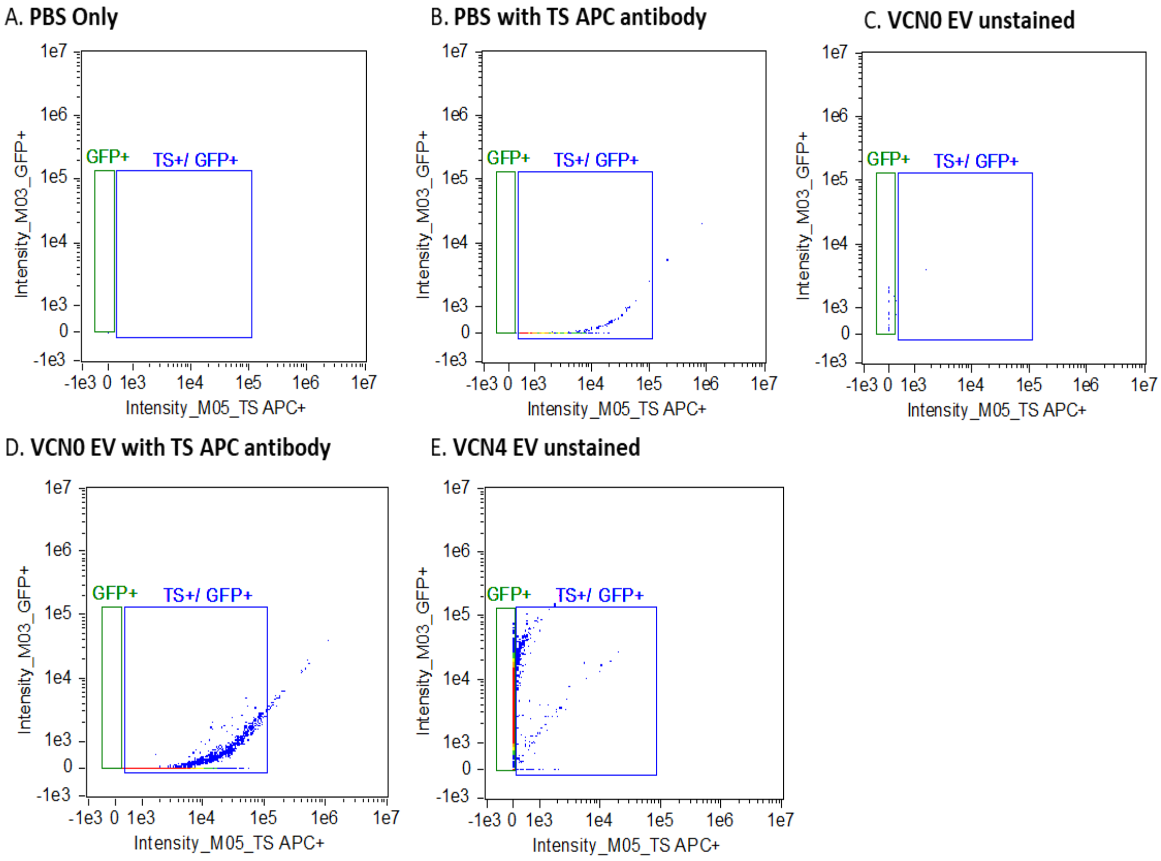

Supplemental Figure S1: Representative control samples for VCN cell-derived EV cargo analysis. Representative IFC analysis of VCN4-derived EVs control samples, including buffer only, antibody only, single-stained EVs, and unstained EVs; **A.** PBS only, **B.** PBS with TS APC antibody, **C.** VCN0 EV unstained, **D.** VCN0 EV with TS APC antibody, **E.** VCN4 EV unstained. (Gating strategy: raw max pixel histogram (not shown; to separate 'SB' events)>intensity (dot plot showing all 'not SB' events (left)).

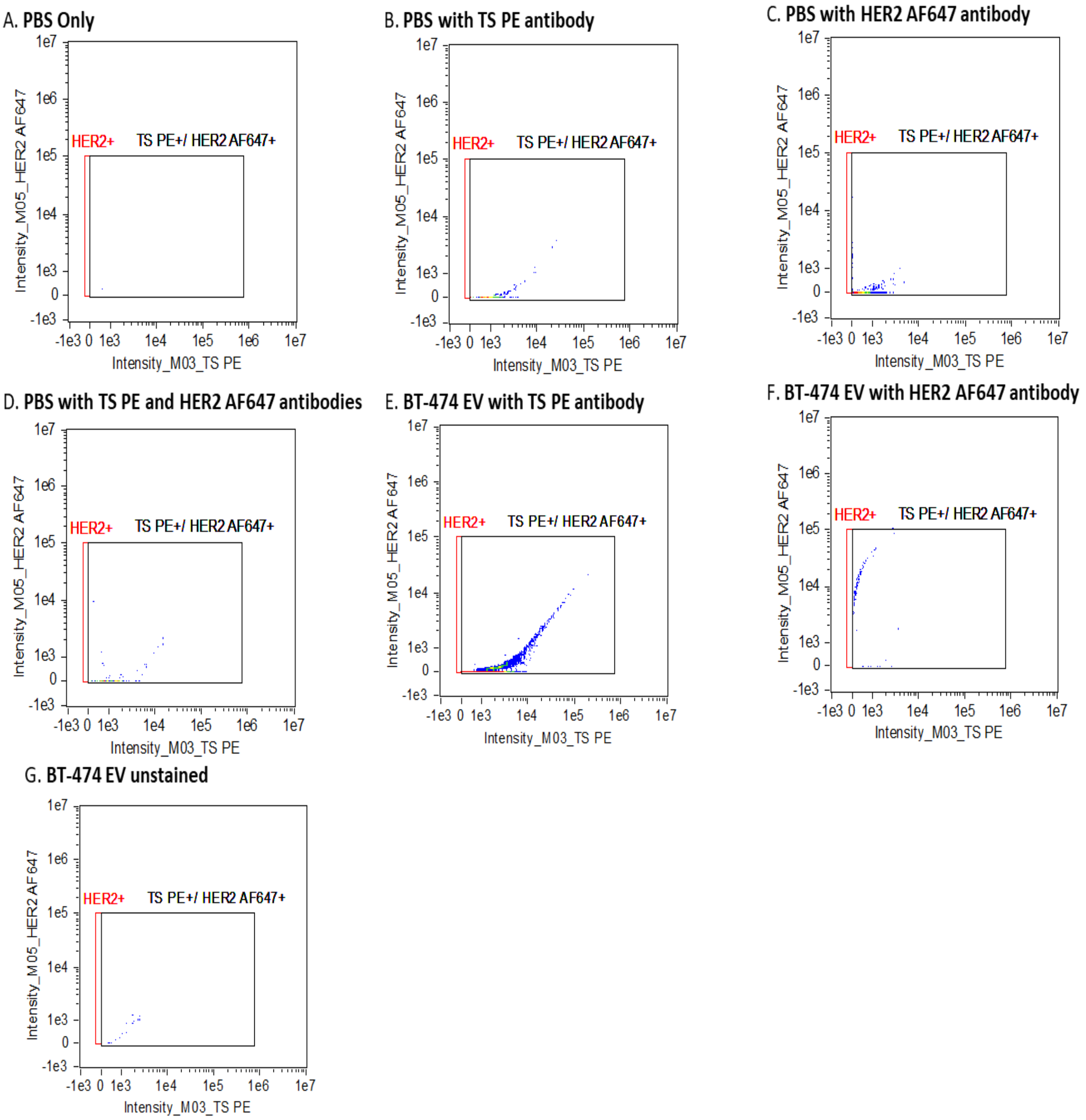

Supplemental Figure S2: Representative control samples for SRM 2373 EV cargo analysis. Representative IFC analysis of BT-474 derived EVs control samples including buffer only, antibody only, EV with FMO, and unstained EV; **A.** PBS only, **B.** PBS with TS PE antibody, **C.** PBS with HER2 AF647 antibody, **D.** PBS with TS PE and HER2AF647 antibodies, **E.** BT-474 EV with TS PE antibody, **F.** BT-474 with HER2 AF647 antibody, and **G.** BT-474 unstained. (Gating strategy: raw max pixel histogram (not shown; to separate 'SB' events)>intensity (dot plot showing all 'not SB' events (left)).
